# COVID-19 and gastrointestinal symptoms in Mexico, a systematic review: does location matter?

**DOI:** 10.1186/s12879-021-06252-y

**Published:** 2021-06-11

**Authors:** Antonio Pizuorno, Nora A. Fierro, Edgar D. Copado-Villagrana, María E. Herrera-Solís, Gholamreza Oskrochi, Hassan Brim, Hassan Ashktorab

**Affiliations:** 1 La Universidad del Zulia, Faculty of Medicine, School of Medicine, Maracaibo, Zulia state 4002 Venezuela; 2 Department of Immunology, Institute of Biomedical Research, National Autonomous University of Mexico, Ciudad University, CP 04510 Mexico City, Mexico; 3 Mexican Social Security Institute, Guadalajara, Mexico; 4 College of Engineering and Technology, American University of the Middle East, Egaila, Kuwait; 5 Department of Medicine, Department of Pathology and Cancer Center, Department of Biochemistry & Molecular Biology, Howard University College of Medicine, 2041 Georgia Avenue, N.W, Washington, D.C, 20060 USA

**Keywords:** Coronavirus disease-19, Pandemic, Gastrointestinal manifestation, Diarrhea, Mexico

## Abstract

**Background:**

Covid-19 in Mexico is on the rise in different parts of the country. We aimed to study the symptoms and comorbidities that associate with this pandemic in 3 different regions of Mexico.

**Methods:**

We analyzed data from SARS-CoV-2 positive patients evaluated at healthcare centers and hospitals of Mexico (*n* = 1607) including Northwest Mexico (Sinaloa state), Southeast Mexico (Veracruz state) and West Mexico (Jalisco state) between March 1 and July 30, 2020. Mexico consists of a total population that exceeds 128 million. Demographics, comorbidities and clinical symptoms were collected. Statistical descriptive analysis and correlation analyses of symptoms, comorbidities and mortality were performed.

**Results:**

A total of 1607 hospitalized patients positive for COVID-19 across all 3 regions of Mexico were included. The average age was 54.6 years and 60.4% were male. A mortality rate of 33.1% was observed. The most common comorbidities were hypertension (43.2%), obesity (30.3%) and diabetes (31.4%). Hypertension was more frequent in West (45%), followed by Northwest (37%) and Southeast Mexico (29%). Obesity was around 30% in Northwest and West whereas an 18% was reported in Southeast. Diabetes was most common in West (34%) followed by Northwest (22%) and Southeast (13%). This might be related to the highest mortality rate in Northwest (31%) and West (37%) when compared to Southeast. Most common symptoms in our overall cohort were fever (80.8%), cough (79.8%), headache (66%), dyspnea (71.1%), myalgia (53.8%), joints pain (50.8%) and odynophagia (34.8%). Diarrhea was the main gastrointestinal (GI) symptom (21.3%), followed by abdominal pain (18%), and nausea/ vomiting (4.5%). Diarrhea and abdominal pain were more common in West (23.1 and 21%), followed by Southeast (17.8, and 9.8%) and Northwest (11.4 and 3.1%).

**Conclusion:**

Our study showed a high mortality rate likely related to high frequencies of comorbidities (hypertension, obesity and diabetes). Mortality was different across regions. These discrepancies might be related to the differences in the frequencies of comorbidities, and partially attributed to differences in socio-economic conditions and quality of care. Thus, our findings stress the need for improved strategies to get better outcomes in our population.

**Supplementary Information:**

The online version contains supplementary material available at 10.1186/s12879-021-06252-y.

## Background

After 7 months of SARS-CoV-2 pandemic, Latin America (LA) is the current epicenter with 8,815,850 diagnosed cases and 325,848 reported deaths as of Sep. 22nd, 2020 [1]. Mexico, the 10th most populated country, with a high poverty rate, major inequalities, and a high prevalence of chronic and metabolic diseases [2], is expected to be one of the most affected countries during the pandemic. According to the evolving data compiled by Johns Hopkins University [3], Mexico has reported more than 700,000 cases and 73,000 deaths as of Sep. 22nd, 2020.

COVID-19 presents primarily as a lower respiratory tract infection, but the multisystemic nature of the disease is commonly found in severe cases. Indeed, a broad spectrum of symptoms associated with COVID-19 has been identified, which range from mild, to moderate to severe symptoms associated with critical illness resulting in respiratory failure or multiorgan dysfunction and/or death. Currently, fever and cough remain the most prevalent symptoms in adults infected by SARS-CoV-2 [4]. Neurological and gastrointestinal symptoms are also encountered [5]. The underlying causes of the variability of COVID-19-related symptomatology and their potential association to different outcomes have not been defined yet. Studies have been conducted in Asian and European populations, the first regions affected by the pandemic. However, differences associated to specific populations present issues with regard to generalizing findings. Evidence supports that human host genetics contribute to the onset of several chronic diseases, including those of infectious nature [6]. This is particularly important in populations with heterogenic heritage such as Mexico, which has an admixture genome [7] differentially distributed across the country [8]. Thus, the study of possible differences in the distribution of COVID-19-related symptoms and comorbidities in distinct regions of Mexico and their effect on disease outcomes need to be evaluated.

Herein, we compared the frequency of COVID-19 related symptoms and comorbidities in patients from 3 different regions of Mexico: Culiacan city at the state of Sinaloa representing Northwest, the state of Jalisco representing West and Veracruz city at the state of Veracruz representing Southeast.

## Methods

### Search Strategy & Selection Criteria

A systematic literature search was carried out of published articles using electronic databases such as PubMed, OVID, Scopus and Google Scholar from March 1st through Jul. 30th, 2020. The following terms were included in the search bar: COVID-19 Mexico. Also, data from the Ministry of Health of Mexico [9] were retrieved.

Inclusion criteria: The following inclusion criteria were used: Confirmed diagnosis of COVID-19 by RT-PCR as reported by the Ministry of Health of Mexico and cross-checked via Johns Hopkins University COVID-19 dashboard. There was no distinction regarding the number of diagnosed cases, sex, age, treatment and/or outcome. Only hospitalized patients were included in this study. Exclusion criteria: Studies where the cases were not confirmed by RT-PCR. studies with incomplete symptoms or comorbidities reports and studies in outpatient settings.

### Study selection

Using the inclusion and exclusion criteria, two papers Villagran-Olivas (ref 10) and Remes-Troche (ref 11) were retained and their detailed patients’ data were retrieved. These two studies had 192 and 112 COVID patients, respectively.

**Fig. 1:**
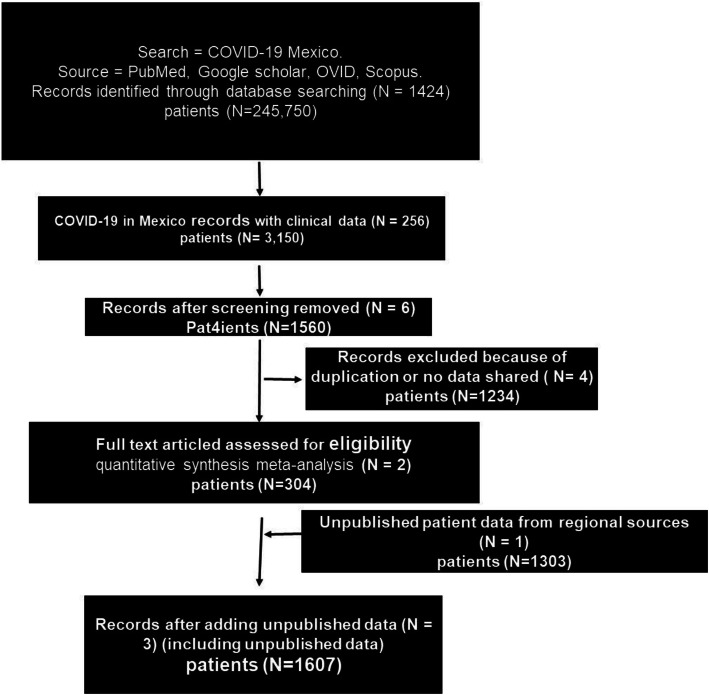
Fellow chart of the study

A third set of data that consists of a retrospective study was also included. We analyzed databases of 1303 SARS-CoV-2 RT-PCR confirmed patients hospitalized at the hospitals of the Delegation of the Instituto Mexicano del Seguro Social (IMSS) Jalisco between March 1st and July 30, 2020. Patients with a confirmatory SARS-CoV-2 test were hospitalized at IMSS based on the CURB-65 scale (confusion, urea, respiratory rate, blood pressure and over 65 years age). Initially, if the patients were COVID-19 positive, and had dyspnea or chest pain, vital signs are immediately taken and if saturation is less than 90%, the patient is hospitalized. The study protocol was approved by the Ethics Committee of the IMSS, Jalisco IRB: 2020–1306-104 (Fig. 1).

### Regions included in Mexico

#### Northwest Mexico

Data from 192 SARS-CoV-2 positive hospitalized patients previously reported [10] admitted between Mar. 1st to May 31st, 2020 at the Hospital Civil de Culiacan, a public hospital located in Culiacan city in the Sinaloa state, were retrospectively analyzed. Culiacan city covers 65 km^2^ (25 sq. mi) and is located at 24°48′25″N 107°23′38″W. It has an elevation of 71 m above sea level (233 ft) and has mean temperature ranges of 70.3 °F, 74.5 °F and 79.5 °F in the months of March, April and May. The population for the city is 858,638 people (166.8 inhab/km^2^).

#### Southeast Mexico

Data from 112 SARS-CoV-2 positive hospitalized patients previously reported [11] admitted between Apr. 1st and May 5th, 2020 at the Hospital Español de Veracruz, a private hospital located in Veracruz city at the Veracruz state, were retrospectively analyzed. Veracruz city covers 90 km^2^ (56 sq. mi), is located at 19°11′25″N 96°09′12″W, has an elevation of 10 m above sea level (30 ft) and has mean temperature ranges of 78.4 °F and 82 °F in the months of April and May, respectively. The population for the city is 609,829 people.

#### West Mexico

A retrospective study was carried out by analyzing databases from West Mexico patients [9] with RT-PCR confirmed SARS-CoV-2 infections of nasopharyngeal swabs. This led to 1303 SARS-CoV-2 patients admitted between Mar. 20th and May 31st, 2020 at the hospitals of the Delegation of the Instituto Mexicano del Seguro Social (IMSS) Jalisco state. The IMSS Jalisco delegation includes 170 medical units, of which 16 are secondary level health care centers and 3 are tertiary level health care centers. The IMSS serves to state formal workers. The state of Jalisco covers 78,599 km^2^ (30,347 sq. mi), is located at 20°34′N 103°41′W, and has an altitude which varies from 0 to 4300 m (o to 14,110 ft). The mean temperature in various regions of the state ranges from 50 to 66.4 °F and in other areas from 66.4 to 71.6 °F. March, April and May are dry months. The population for the state is 7,844,830 (100 inhab/km^2^).

#### Systematic analysis

Tables were generated from the retrieved data. The tables included the following information: report date, location, confirmed cases, deaths, median age, symptoms (headache, cough, myalgia, fever, odynophagia, anosmia, dyspnea, ageusia, thoracic pain, tachypnea, cyanosis, nasal congestion, joint pain and fatigue, diarrhea, abdominal pain, nausea and vomiting), comorbidities (hypertension, diabetes, asthma, obesity, cardiovascular disease, COPD, immunosuppression, cancer, IBD). Body Mass Index (BMI) was calculated based on a person’s weight in kilograms divided by the square of height in meters. Any person with BMI more than 25 Kg/M2 and less than 30 is overweight and those > 30 are considered obese. This study used the American Diabetes Association 2020 Criteria for Diabetes Mellitus diagnosis, patients with a A1C higher or equal to 6.5%, Fasting plasma glucose higher or equal to 126 mg/dl are considered diabetic. Our cohort database only included patients medical histories (diabetes, hypertension,..) but no medication history.

### Statistical analysis

Patient demographics, symptoms, underlying comorbidities and mortality rates were compared in hospitalized patients from Northwest, West and Southeast Mexico. The common symptoms and comorbidities were combined and analyzed by weighted analysis methods where applicable. Correlation coefficients were calculated, where applicable, to establish associations between comorbidities and mortality. Mortality, also known as Case Fatality Rate correspond to total of dead COVID-19 patients/total COVID-19 patients (× 100). The effect of symptoms was reported using weighted analysis where weights were related to the size of the reported study. SPSS version 26 (SPSS Inc., Chicago, IL, USA) was used for this analysis.

## Results

### High mortality in hospitalized patients in Mexico

To determine if COVID-19 outcome in Mexico was as reported worldwide, the study population from Northwest, West and Southeast regions was analyzed. A total of 1607 confirmed hospitalized cases were included. According to the overall weighted average analysis, the average age was 54.6 years (+SD 3.4_) and the percentage of distribution between males and females was 60.4% versus 39.4%, respectively. A high mortality rate of 33.1% was observed **(**Table 1**)**. The mortality rate for hospitalized and ambulatory cases reported by the Ministry of Health in Mexico for the same time period was 10–12% [9].

**Table 1:** Demographic characteristics, mortality, symptoms and comorbidities in hospitalized COVID-19 Mexican patients

Characteristics	Percentage
Patient n = 1607	%
Gender (male)	60.4
Mortality	33.1
Symptoms	
Fever	80.8
Cough	79.8
Headache	66
Fatigue	56.2
Dyspnea	71.1
Myalgias	53.8
Joint pain	50.8
Odynophagia	34.8
Nasal congestion	10.4
Tachypnea	8.6
Thoracic pain	33.2
Anosmia	5.9
Ageusia	6.1
Cyanosis	6.7
Hypertension	43.2
Obesity	30.3
Diabetes	31.4
COPD	6.5
CV disease	4.4
Immunocompromised	3.3

**Table 2:** COVID-19-associated symptoms in hospitalized patients from distinct regions of Mexico

Symptoms/Region (%)	Northwest (***n*** = 192)	West (***n*** = 1303)	Southeast (***n*** = 112)
Headache (%)	74.8	65.4	58.9
Cough (%)	83.8	79.2	80.3
Myalgias (%)	29.1	56.4	66.9
Fever (%)	84.4	79.8	86.6
Odynophagia (%)	15.6	38	31.2
Anosmia (%)	NR	5.9	7.1
Dyspnea (%)	65.1	75.9	25.6
Ageusia (%)	NR	6.1	7.1
Thoracic pain (%)	5.7	38.9	15.1
Tachypnea (%)	16.6	7.5	NR
Cyanosis (%)	2	7.5	NR
Nasal congestion (%)	10.4	NR	NR
Joint pain (%)	22.9	55	NR
Fatigue (%)	56.2	NR	NR

### Comorbidities in hospitalized COVID-19 Mexican patients

**Fig. 2:**
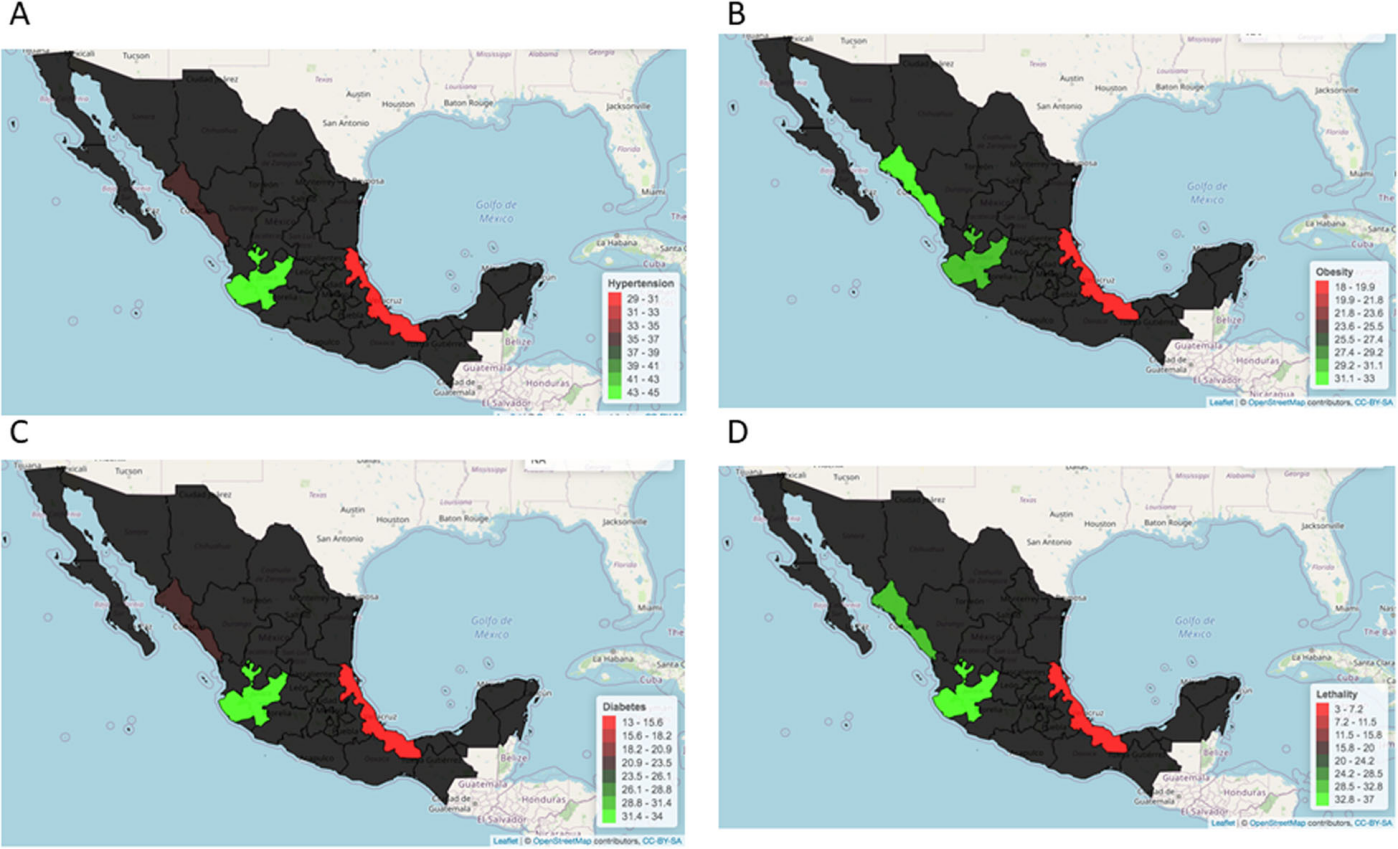
Differential frequencies in hypertension (**A**), obesity (**B**), diabetes (**C**) and mortality (**D**) in Northwest, West and Southeast COVID-19 Mexican Patients (figure is our own work)

The most common comorbidities in our cohort were hypertension with 43.2%, followed by obesity with 30.2% and diabetes with 31.4% (Fig. 2). As for mortality rate, the prevalence of these comorbidities was higher than previously reported by Giannouchos et al. (I think we should eliminate this and refrain to make any discussion and/or comparison of the results with other references in the results section). in a large cohort of 87,756 COVID-19 patients from Mexico (20.5% for hypertension, 20.9% for obesity and 17.5% for diabetes) [12]. These differences might be related to the fact that the above-mentioned report includes hospitalized and ambulatory patients in its analysis and only hospitalized patients were included in our study.

### Comorbidities and mortality in hospitalized patients vary in northwest, west and Southeast Mexico

We conducted an analysis to characterize plausible differences in COVID-19 behavior in Northwest relative to West and Southeast Mexico. As in the overall analyzed group, hypertension, obesity and diabetes were the most frequent underlying comorbidities in the three regions when they were independently analyzed. However, differences in the comorbidities frequencies were found when regions were compared. Hypertension was most frequently found in West (45%), followed by Northwest (37%) and Southeast (29%). Obesity was around 30% in Northwest and West whereas an 18% was reported in Southeast. Diabetes was most common in West (34%) followed by Northwest (22%) and Southeast (13%). This data underscores common frequencies in comorbidities in Northwest and West Mexico relative to Southeast **(**Fig. 2**).** This might be related to the highest mortality rate in Northwest (31%) and West (37%) when compared to Southeast (3%) **(**Fig. 2**).**

### Symptoms in hospitalized Mexican patients

According to the overall weighted average for symptoms, and as reported worldwide [13], the most common symptoms were fever (80.8%) followed by cough (79.8%), headache (cephalea; 66%), dyspnea (71.1%), myalgia (53.8%), joint pain (50.8%) and odynophagia (34.8%) (Table 1). As previously reported in South Mexico [11], overall weighted average analysis underscored that diarrhea was overall the main gastrointestinal symptom (21.3%), followed by abdominal pain (18%), nausea and vomiting (4.5%). The prevalence of diarrhea and abdominal pain was similar to a previous report from a meta-analysis including data from patients in the United States [14]. Anosmia (5.9%) and ageusia (6.1%) were also found in our analyzed population (Table 1). In Addition, symptoms analysis was performed using weighted method (Suppl. Table 1).

In our analysis, fever, cough and headache were equally distributed between regions. Myalgia was less common in North (29.1%) relative to West (56.4%) and Southeast (66.9%). Odynophagia was most common in West (38%) and Southeast (31.2%) than in Northwest (15.6%). Thoracic pain was more common in West (38.9%), followed by Southeast (15.1%) and Northwest (5.7%). Dyspnea was more common in West (75.9%), followed by Northwest (65.1%) and Southeast (25.6%) (Table 2).

### Gastrointestinal symptoms were differentially distributed in distinct regions

Reported gastrointestinal (GI) symptoms were differentially distributed among COVID-19 patients in distinct regions of Mexico. Diarrhea was the main GI symptom in the analyzed regions. However, its frequency was different across regions. Diarrhea and abdominal pain were more common in West (23.1 and 21%), followed by Southeast (17.8, and 9.8%) and Northwest (11.4 and 3.1%). Nausea and vomiting were more frequently found in Southeast (7.1%) than in Northwest (3.1%), and they were not reported in West (Table 3). We also did the gastrointestinal analysis using weighted method (suppl. Table 2).

**Table 3:** COVID-19-associated gastrointestinal symptoms in hospitalized patients from distinct regions of Mexico

Symptoms/Region (%)	Northwest (n = 192)	West (n = 1303)	Southeast (n = 112)
Abdominal pain (%)	3.1	21	9.8
Diarrhea (%)	11.4	23.1	17.8
Nausea and vomiting (%)	3.1	NR	7.1

## Discussion

According to the evolving data compiled by Johns Hopkins University [3], Mexico is one the top 10 countries in the world with the highest number of COVID-19 associated deaths (accessed on Sep. 22nd, 2020). Underlying comorbidities including metabolic syndrome diseases such as diabetes, hypertension, obesity and cardiovascular diseases represent unfavorable factors for positive COVID-19 outcome. Indeed, from a cohort of 177,133 confirmed COVID-19 cases in Mexico, Bello-Chavolla et al. recently reported that obesity mediates around 50% of the effect of diabetes on COVID-19 mortality [15] and in an independent study of 212,802 confirmed SARS-CoV-2 cases in Mexico, Hernandez-Galdamez et al. reported that noncommunicable diseases underlying comorbidities increase the risk of severe disease [16]. As such, Mexico is currently in the middle of two major public health threats: obesity and SARS-Cov2 infection. According to the Ministry of Health in Mexico, chronic diseases represent 7 of the 10 main causes of death. The most relevant are diabetes and heart disease, both closely related to obesity. The national prevalence of diabetes and hypertension in Mexico are 10.3 and 18.4%, respectively and the prevalence of overweight and obesity in adults older than 20 years old is 75.2%. Obesity, alone, in older than 20 years old accounts for 36.1% [2].

In this retrospective cohort study, we analyzed data from COVID-19 patients from Northwest, West and Southeast Mexico. Globally, a high mortality rate of 33.1% was observed. This may be related to the fact that only data from hospitalized patients were analyzed in the present study. It is also important to note that COVID-19-related hospitalization protocols in Mexico prioritize the admission of severe cases. This high mortality is in agreement with current reports puting Mexico among countries with the highest mortality rates associated to COVID-19 worldwide [3]. Since the beginning of the pandemic, the mortality rate reported by the Ministry of Health in the country ranged from 10 to 12% (including hospitalized and ambulatory patients), still a higher rate than what is reported worldwide (3–4%). Thus, our finding stress the need to improve strategies to get better outcomes in Mexican population.

Interestingly, our data showed a high mortality rate in Northwest and Western regions relative to Southeast. These discrepancies might be related to the differences in the frequencies of hypertension, obesity and diabetes. These comorbidities were more frequently found in Northwest (37, 30 and 22%, respectively) and West (45, 30 and 34%, respectively) relative to Southeast Mexico (29, 18 and 13%, respectively). The Ministry of Health data however reports that the prevalence of hypertension in Jalisco (West Mexico) as 14%, in Sinaloa (Northwest) as 16.2%, and in Veracruz (Southeast) as 23.6%. Obesity prevalence as reported by the Ministry of Health in Jalisco, Sinaloa, and Veracruz is 12.9, 15.8, and 22.9%, and the Diabetes prevalence in these states is 7.6, 10.7, and 11.9%, respectively [17]. The differences in the frequencies of comorbidities in our study compared to the data reported by the Ministry of Health might be related to the fact that official data include general population and a large number of individuals when compared to our study population. Moreover, the differences in mortality and comorbidities when regions were independently analyzed in our study may partially be attributed to differences in socio-economic conditions and quality of care, given patients attending to a private hospital in Southeast Mexico showed lower mortality rate and reduced frequency in comorbidities relative to patients either attending to public hospitals (Northwest) or hospitals serving state formal workers (West).

According to indicators published in 2018 that include education levels, access to health services, access to social security, access to food, quality of housing spaces and access to basic services in housing, the National Council to the Evaluation of Social Policy [18] estimated that 41.9% of the population in Mexico is in a situation of poverty. Therefore, it is necessary to prioritize health care services to serve the most vulnerable populations including those in conditions of poverty.

As reported worldwide, fever, cough and headache were the most common symptoms in the cohort. These symptoms were equally distributed between regions. However, slight differences in the frequencies of myalgias and odinophagia were found, with less reported cases in Northwest compared to West and Southeast. Overall, diarrhea was the main gastrointestinal symptom; anosmia and ageusia were also reported. Interestingly, subgroup analyses underscored differences in the frequencies of gastrointestinal symptoms when regions were independently analyzed. Diarrhea and abdominal pain were more common in West, followed by Southeast and Northwest. Taking into account that data from Northwest corresponded to patients attending public hospitals, differences in gastrointestinal symptoms do not seem to be associated with socioeconomic status. However, a larger number of cases need to be analyzed in order to demonstrate this possibility. Moreover, additional factors should be considered to explain the differences in the distribution of gastrointestinal symptoms between regions. In this sense, as in most Latin American countries, the genetic structure of the Mexican population is an admixture of three paternal lineages consisting of Amerindian, European and African ancestry [7]. European and Amerindian ancestry prevail in North and West Mexico, while African and Amerindians prevail in East Mexico. In addition, there are still nearly 15 million Native Amerindians who still maintain their inherited traditions, living across the country [19]. Taking into account that factors including host genetics may affect infectious disease behavior [20], the differences in gastrointestinal symptoms may be related to ethnic composition. Genetic background may also be related to differences in mortality and comorbidities when regions were independently analyzed. Thus, detailed analysis of COVID-19 epidemiologic status in a large number of regions with similar genetic background and different socio-economic status is required to assess the roles these confounders play in observed symptoms and outcomes.

Limitation of our study include: 1) overall sample size, 2) distinct sample size between regions, 3) possible selection bias given that data correspond to patients from public and private hospitals in distinct proportions, 4) possible selection bias given the protocols in Mexico prioritize the hospitalization of severe cases.

## Conclusion

The emergency imposed by COVID-19 pandemic highlights the need for better notification and recording systems to estimate the real impact of the disease behavior globally. Close monitoring of underlying comorbidities, symptoms and outcomes is recommended in the construction of predictive models to determine risk populations for infection and poor outcomes. Joint efforts in low-income countries as in most of Latin America are needed to assess the disease behavior and to lower its impact. Given similar economic, socio-demographic, environmental and ethnic conditions prevail in Latin America, our findings may benefit the entire region. However, future studies are required to determine the specific strategies to handle the pandemic at local levels.

## Supplementary Information

**Additional file 1 **Suppl. Table **1**: Symptoms analysis was performed using weighted method. Suppl. Table 2**.** Gastrointestinal analysis was performed using weighted method.

## Data Availability

All weighted de-identified data used here is included in this study.

## Abbreviations

ACE2: Angiotensin Receptor 2
CI: Confidence Interval
COVID-19: Coronavirus Disease-19
GI: Gastrointestinal
RT-PCR: Real-time Polymerase Chain Reaction
SARS-CoV-2: Severe Acute Respiratory Syndrome Coronavirus 2
