# Supplementary material for: COVID-19 and gastrointestinal symptoms in Mexico, a systematic review: does location matter?
Source: BMC Infect Dis. 2021 Jun 11;21:555. doi: 10.1186/s12879-021-06252-y (PMC8193163; doi:10.1186/s12879-021-06252-y)
Supplement: Supplementary file 1 — Additional file 1 Suppl. Table 1: Symptoms analysis was performed using weighted method. Suppl. Table 2. Gastrointestinal analysis was performed using weighted method. [file 12879_2021_6252_MOESM1_ESM.docx]

Suppl. Table 1: Symptoms analysis was performed using weighted method

| **Case Summaries^a^** | | | | | | | | | | | | | | | | | |
| --- | --- | --- | --- | --- | --- | --- | --- | --- | --- | --- | --- | --- | --- | --- | --- | --- | --- |
|  | | | | Cephalea In % | Cough | Mialgyas | Fever | Odinophagia | Anosmia | Dyspnea | Ageusia | Thoracic pain | Taquipnea | Cyanosis | Nasal congestion | Joint pain | Fatigue or tiredness |
| Country | Mexico East | 1 | | 58.900 | 80.300 | 66.900 | 86.600 | 31.200 | 7.100 | 25.600 | 7.100 | 15.100 | . | . | . | . | . |
|  |  | Total | N | 112 | 112 | 112 | 112 | 112 | 112 | 112 | 112 | 112 |  |  |  |  |  |
|  | Mexico North | 1 | | 74.800 | 83.800 | 29.100 | 84.400 | 15.600 | . | 65.100 | . | 5.700 | 16.600 | 2.000 | 10.400 | 22.900 | 56.200 |
|  |  | Total | N | 192 | 192 | 192 | 192 | 192 |  | 192 |  | 192 | 192 | 192 | 192 | 192 | 192 |
|  | West Mexico | 1 | | 65.400 | 79.200 | 56.400 | 79.800 | 38.000 | 5.900 | 75.900 | 6.100 | 38.900 | 7.500 | 7.500 | . | 55.000 | . |
|  |  | Total | N | 1303 | 1303 | 1303 | 1303 | 1303 | 1303 | 1303 | 1303 | 1303 | 1303 | 1303 |  | 1303 |  |
|  | Total | N | | 1607 | 1607 | 1607 | 1607 | 1607 | 1415 | 1607 | 1415 | 1607 | 1495 | 1495 | 192 | 1495 | 192 |
|  | | | | | | | | | | | | | | | | | |

**Suppl. Table 2.** Gastrointestinal analysis was performed using weighted method

| **Case Summaries^a^** | | | | | | |
| --- | --- | --- | --- | --- | --- | --- |
|  | | | | Abdominal pain | Diarrhea | Nausea and Vomits |
| Country | Mexico East | 1 | | 9.800 | 17.800 | 7.100 |
|  |  | Total | N | 112 | 112 | 112 |
|  | Mexico North | 1 | | 3.100 | 11.450 | 3.100 |
|  |  | Total | N | 192 | 192 | 192 |
|  | West Mexico | 1 | | 21.000 | 23.100 | . |
|  |  | Total | N | 1303 | 1303 |  |
|  | Total | N | | 1607 | 1607 | 304 |
|  | | | | | | |
